# Development of molecular detection methods of *Bovicola ovis* from sheep fleece

**DOI:** 10.1007/s00436-022-07520-9

**Published:** 2022-04-18

**Authors:** Lily Tran, Grant T. Rawlin, Travis Beddoe

**Affiliations:** 1 Department of Animal, Plant and Soil Sciences, School of Life Sciences, La Trobe University, Bundoora, VIC 3083 Australia; 2 AgriBio, Centre for AgriBioscience, Bundoora, VIC 3083 Australia; 3 Department of Jobs, Precincts and Regions, Agriculture Victoria Research, Bundoora, VIC 3083 Australia

**Keywords:** Sheep, qPCR, Molecular detection, Parasitology, LAMP, *Bovicola ovis*, Isothermal amplification, Lice

## Abstract

The sheep body louse (*Bovicola ovis*) commonly referred to as sheep lice are small chewing ectoparasites of sheep. Infection results in significant economic costs to the Australian sheep industry due to reduced wool quality caused by chronic itching from sheep rubbing and biting fleece. Treatment relies on use of insecticides; however, resistance has developed against pyrethroid and other insect growth regulator lousicides. There is urgent need to develop cost-effective lice management to reduce the use of insecticides, with the application of insecticidal treatments only applied when an infestation is detected. However, the current detection method relies on fleece parting for detection of *B. ovis* which is highly dependent on the skill of the inspector, the number of sheep examined, and the prevalence and severity of the infestation. To improve *B. ovis* detection, a highly sensitive (5 × 10^−8^ ng/μL) and specific multiplex quantitative PCR which simultaneously detects sheep lice and sheep DNA was developed. In addition, a *B. ovis* loop-mediated isothermal amplification (LAMP) assay was developed for field use. The *B. ovis* LAMP (Bov-LAMP) assay was optimized to reliably detect *B. ovis* from wool samples down to 5 × 10^−6^ ng/μL, with time to positive (T_p_) < 10 min. Both assays demonstrate high sensitivity and specificity, enabling rapid identification of *B. ovis* DNA from sheep fleece samples and have the capacity to be used for ongoing management and surveillance of *B. ovis* in Australian sheep flocks.

**Supplementary Information:**

The online version contains supplementary material available at 10.1007/s00436-022-07520-9.

## Introduction

Australian sheep flocks are intensively farmed for meat and wool, with wool contributing an estimated AUD $2.6 billion to the Australian economy (James 2010). Wool quality is adversely affected by the ectoparasite *Bovicola ovis* (previously *Damalinia ovis*), commonly referred to as sheep lice (Niven and Pritchard 1985). Louse infestations are spread through direct contact, causing fleece derangement from chronic itching due to lice feeding on scurf and wool yolk, thereby causing sheep to rub and bite their fleece. This irritation reduces wool quality, costing in an estimated AUD $123 million in losses annually, including treatment and control costs of this parasite (Horton and Carew 2014).

Epidemiological prevalence on this parasite is currently uncertain, with estimates of up to 25% prevalence in Australian sheep (Popp et al. 2012). Lack of prevalence data is largely due to no rapid detection methods, with current detection relying on visual fleece inspection, parting fleece to manually count lice based on suspected lousy sheep displaying rubbing symptoms, and a minimum 10 cm area of fleece parted to observe for lice. The process is repeated either 20 times, or until a single live louse is found (Horton et al. 2015). This is time consuming and lacks sensitivity thereby missing low level infections as sheep lice are <2 mm in length (Lucas et al. 2017b). This issue is further compounded as visible infections are slow to develop, with most infestations not visibly detectable before 3–6 months post infection. It is also estimated that the detection of one louse per 10 cm fleece parting suggests that the sheep has an indicative infestation of 3000–4000 lice (James 2010). Visual detection methods are further complicated with routine administration of insecticides intended to eradicate infestation. Incorrect application by under-dosing, not rotating actives, failing to maintain jetting and/or dipping equipment further hinder detection methods as improper treatment use drastically reduces population numbers, however fails to eliminate the infection resulting in population numbers gradually returning to pre-treatment levels, in addition to contributing to increasing prevalence of drug-resistant lice (McLeod 1995).

Lice detection is important, where individual identification is critical for sale yards, stray sheep, and drug-resistance screening, as Australian legislation and farming practices for the management of lousy sheep vary by state with financial penalties incurred by presenting lousy sheep at sale yards (Lucas et al. 2017a). Despite this, penalties are seldom enforced due to a lack of convenient testing alternatives. One way to mitigate the lack of molecular diagnostics is to utilize nucleic acid amplification tests (NAATs) frequently used for pathogen screening (Amoah et al. 2017). The most used NAATs are PCR-based assays which are routinely used as reference assays for disease confirmation of pathogens (Hoffmann et al. 2009; Wang et al. 1997; Yang and Rothman 2004).

Of the NAATs available, an alternative to PCR is loop-mediated isothermal amplification (LAMP). This method of target DNA amplification uses a strand-displacing polymerase and four to six primers which serve to separate double stranded DNA, initiating loop formation which provides additional primer recognition sites for rapid amplification. Combined, these parameters eliminate thermocycling requirements that are a major constraint of PCR-based amplification. This has enabled field and low-resource deployment of LAMP using water-baths, heat-blocks and even a thermos for pathogen detection (Barkway et al. 2011; Bath et al. 2020; Gandasegui et al. 2016; Nagamine et al. 2002; Notomi et al. 2000; Poole et al. 2017). Considering the restraints of PCR amplification, LAMP has the capacity to fulfill the requirements for rapid testing without requiring a central laboratory. Amplification is generally completed within an hour, with LAMP studies reporting high sensitivity of detection limits in picogram ranges, capable of detecting low-level infections (Noden et al. 2018).

Given the current state of diagnostics for *B. ovis* and that NAATs are rapid and low-cost relative to traditional immunological assays, two NAATs were developed for the detection of *B. ovis* to further improve molecular detection methods*.* Firstly, a qPCR assay was developed and used as a reference assay for subsequent LAMP optimization. Secondly, the *B. ovis* LAMP was optimized to reduce time to results. These assays were developed for the detection of *B. ovis* since the withdrawal of the on-farm ELISA (Popp et al. 2012).

Molecular assays for the detection of *B. ovis* have recently been developed which aim to address the current issues surrounding lice detection and improve diagnostic capacity (Wong et al. 2020). Two assays were developed utilizing PCR and LAMP which reduced labor required for sheep constraint compared to visual detection, however had variable analytical performance with low specificity, frequently recording false positive and/or negative results. The aim of this study was to develop a qPCR assay as a reference assay intended to reduce manual inspection of lousy wool, and to validate *B. ovis* LAMP detection from fleece through comparison against the *B. ovis* qPCR and visual inspection references. Further, the *B. ovis* LAMP was additionally assessed for suitability in field-based settings using a crude extraction method to further reduce time-to-results.

## Methods

### Samples and DNA extraction

Sheep fleece including tailings were provided by farmers from various locations in South Australia and Victoria, Australia. Samples were obtained from farming properties with suspected lousy sheep based on farmer observations and veterinary records, or from known negative properties. Five-hundred-gram post satchels were provided, and participants instructed to place approximately 30 g of fleece per sheep into each satchel. Satchels were then posted to AgriBio Center for AgriBiosciences, Bundoora, Victoria, and stored at −20 °C until processing. Details of each sample used in this study are provided in Table 1.

**Table 1:** Submitted fleece samples used for *B. ovis* qPCR and LAMP development. Fleece samples were sent to from properties in South Australia (SA) and Victoria (Vic), classified as positive (+) or negative (−) for *B. ovis* based on submitter observations

Sample code	Location	Reported lice infection status
VIC01	Vic	+
VIC02	Vic	+
VIC03	Vic	−
VIC04	Vic	−
VIC05	Vic	−
VIC06	Vic	−
VIC07	Vic	−
SA01	SA	+
SA02	SA	+
SA03	SA	+

Total DNA from the samples was extracted using a combination of a modified table locks test, DNA precipitation, and environmental DNA extraction methods (Czeglédi et al. 2021; Djurhuus et al. 2017; Li and Sheen 2012; Morcombe et al. 1996; Rathinasamy et al. 2018). The table locks test was modified by dissolving 10 g of wool washed three times in 1% (v/v) Triton x-100 to reduce lanolin lipids, excess water removed, then placed in 150 mL 10% (w/v) NaOH on a stirring heat-block at 90 °C for 1 h, or until all wool was dissolved. Reported lice positive samples were briefly examined during the weighing process for lice. If a louse was found, the sample was considered positive for *B. ovis*. The resulting solution was cooled to ambient temperature, then filtered through a 1 mm mesh filter to remove large debris and further strained using a 70 μm EASYstrainer cell sieve (Greiner, Kremsmünster, Austria). A 10 mL aliquot was taken from each sample and centrifuged at 4000 × *g* for 5 min to separate the mixture into an upper layer containing residual lipids, a middle aqueous phase containing DNA, and sediment containing fine organic matter, including dirt. Samples were transferred into fresh 50 mL conical tubes, taking care not to transfer the upper and sedimented layers, containing 1:10 vol 3 M sodium acetate, 2.5 vol pre-chilled absolute ethanol, and incubated at −20 °C overnight or −80 °C for 2 h. Samples were pelleted by centrifugation at 4700 × *g* for 40 min and the supernatant discarded. The DNA pellet was re-suspended in 500 μL nuclease free water (NFW) and transferred to a fresh microfuge tube containing 2 vol 6 M sodium iodide and 100 μL 100 mg/mL silicon dioxide (SiO_2_). Tubes were foiled then placed on a tube rotator at low speed for 1 h to facilitate DNA binding.

Samples were then pelleted by centrifugation, the supernatant discarded, and the pellet resuspended in 500 μL DNA wash buffer (50% (v/v) ethanol, 10 mM Tris-HCl pH 7.5, 100 mM sodium chloride, 1 mM EDTA). Samples were re-pelleted by centrifugation, and the wash process repeated a further two times. After three washes, the supernatant was discarded, SiO_2_ re-pelleted, then dried at 70 °C for 1 min to evaporate residual ethanol. Samples were resuspended in 30 μL NFW and incubated at 70 °C for 2 min to elute before being centrifuged at 15,000 × *g* for 1 min to pellet the SiO_2_ and the eluate transferred to a fresh microfuge tube and stored at −20 °C until required. Fleece DNA extraction was confirmed by qPCR amplification using universal *Ovis aries cytochrome c oxidase subunit 1 mitochondrial gene* (COI) primers described below.

### Sample preparation using minimal processing methods

All fleece samples (Table 1) were swabbed to evaluate rapid, field appropriate sample preparation. Sterile rayon fiber swabs (CLASSIQSwabs™, Copan, Mantua, Italy) were run through all 10 g portions of fleece. Swabs were slowly rotated through fleece with particular attention given to the base of the wool shaft if present. Swab heads were then placed into microfuge tubes containing 500 μL 10% w/v Chelex-100 resin (Bio-Rad, CA, USA) prepared in NFW, briefly stirred, before snapping swab handles against the side of the tube prior incubation at 90 °C—10 min. Samples were cooled to ambient temperature, allowing the resin to sediment prior to *B. ovis* LAMP amplification.

### Preparation of *Bovicola ovis* standards

All assays were optimized using a synthetic construct containing the full *B. ovis* COI sequence (GenBank Accession #: MH001203.1). A 1326 bp amplicon was amplified by PCR targeting nucleotide positions 75-1400 containing the gene coding sequence. PCR amplification was carried out in 50 μL reaction volumes consisting of 1× *Pfu* DNA polymerase buffer with MgSO_4_ (Promega, Madison, USA), 200 μm ea dNTPs, 0.5 μm each primer (Bov_3_20 5′-ACGATGGGTAGGTTCAAC-3′ and Bov_1311_1328 5′-ACGTCTGGGTAATCACAG-3′), 1.25 U *Pfu* DNA polymerase, 0.1 μg synthetic construct, and made up to 50 μL with NFW. Amplification was carried out under the following cycling conditions: initial denaturation 95 °C for 2 min, followed by 35 cycles of 95 °C for 1 min denaturation, 62 °C for 30 s anneal, 72 °C for 3 min extension, with a final extension of 72 °C for 5 min. Products were separated on a 1% (w/v) agarose gel in 0.5× Tris-borate EDTA stained with 0.5 μg/mL EtBr and visualized on a Bio-Rad Gel Doc (Bio-Rad, CA, USA). Products were purified using a FastGene Gel/PCR Extraction Kit (NIPPON Genetics, Tokyo, Japan) per manufacturer instructions and eluted twice in 30 μL NFW. DNA was quantified by Qubit dsDNA BR and sent for Sanger Sequencing to confirm correct sequence amplification at The Australian Genome Research Facility (Victoria, Australia). Products were standardized to 5 ng/μL, and ten-fold serial dilutions prepared from 5 × 10^−3^ to 5 × 10^−9^ ng/μL in TE buffer (1 mM Tris-HCl, 0.1 mM EDTA pH [8.0]) and stored at −20 °C until required.

### Primer design

Quantitative PCR primers were designed using NCBI Primer BLAST (https://www.ncbi.nlm.nih.gov/tools/primer-blast/), targeting the same *B. ovis* COI sequence previously detailed, amplifying a 108 bp region (Supplementary Table 1). A universal sheep COI assay obtained from Parker et al. (2020) was included and multiplexed with the *B. ovis* qPCR as an endogenous positive extraction control to confirm successful DNA extraction from dissolved fleece samples (Supplementary Table 1). LAMP primers were designed to target the same COI sequence using Primer Explorer V5 (Eiken Chemical Company; https://primerexplorer.jp/e/) with default settings (Table 2, Fig S1). Each *B. ovis* primer was initially assessed for specificity in silico through NCBI BLAST (https://blast.ncbi.nlm.nih.gov/Blast.cgi).

**Table 2:** Loop-mediated isothermal amplification primers used for the detection of *B. ovis*

Primer name	Sequence 5′-3′	Nucleotide position
Bov_3_F3	AGTCAACCTGGTTATTCAGT	400
Bov_3_B3	GGAAGGGATAGAAGGAGAAG	571
Bov_3_FIP	AGAGCAAATAAAGTTAATTGCCCCTGATCTATCGATCTTTTCTTTACACC	F2: 412 F1c: 468
Bov_3_BIP	GCCCTTAACCTTTGGGTCGAGACAGCTGTAATAAGAACCG	B2: 551 B1c: 493
Bov_3_LF	ATAATCCTCCTAACCCCAGCCA	446
Bov_3_LB	GACGTGATAATGTTGTTTTGCTGAG	526

### *Bovicola ovis* qPCR assay optimization

Quantitative PCR conditions were optimized using previously prepared standards. During initial assay development, different primer concentrations and cycling conditions were assessed, with final optimized reactions carried out in 20 μL volumes consisting of 1× SensiMix Probe No-ROX master mix (Meridian Bioscience, OH, USA), 0.4 μm primers and 0.1 μm probe for *B. ovis* (Supplementary Table 1), 0.5 μm primers and 0.25 μm probe for *O. aries* (Supplementary Table 1), 2 μL template DNA, and adjusted to 20 μL with NFW. Amplification was carried out in a MIC qPCR cycler (BioMolecular Systems, Queensland, Australia) with activation at 95 °C for 10 min, followed by three-step amplification with 40 cycles of 95 °C for 15 s denaturation, 62 °C for 15 s anneal, and 72 °C for 15 s extension, with data acquisition on green and red channels. Standard curve data was analyzed using the MIC PCR program (V2.6.4) using the bulk analysis function. All qPCR runs were analyzed using dynamic normalization method with the first five amplification cycles excluded, and a set threshold of 0.5 normalized fluorescence units.

### *Bovicola ovis* qPCR validation

All reactions were performed in duplicate, with a standard curve of *B. ovis* COI standards ranging 5 × 10^−3^–5 × 10^−9^ ng/μL run for quantification, serving as positive controls to assess inter- and intra-assay variation and a no template control (NTC) to monitor contamination. Additionally, the standard curves were used to assess assay sensitivity through determining assay limit of detection (LoD). Runs were excluded and repeated if the cycle quantification (Cq) standard deviation of any standards exceeded 0.5 from historical averages. Fleece samples prepared earlier (Table 1) were assessed using different dilution ranges, and a dilution factor of 1/10 was chosen as some samples failed to amplify undiluted on initial validation runs.

Fleece DNA extraction was considered successful if samples returned positive Cq values for *O. aries*, samples that failed to qPCR amplify for *O. aries* were repeated and further diluted 1/20. Samples were considered positive for *B. ovis* if both replicates amplified. Additionally, a bacterial specificity panel consisting of species commonly found in sheep environments was prepared. Total genomic DNA from these samples was extracted using a Bioneer AccuPrep Genomic DNA extraction kit following manufacturer instructions for gram positive and negative bacterial preparations, eluted in 100 μL EA buffer and standardized to 5 ng/μL prior to storage at −20 °C until use (Table 3). Any samples that had unexpected amplification were repeated.

**Table 3:** Bacterial specificity panel used to validate the *B. ovis* qPCR and LAMP assays

Species	Abbreviation	Source	Strain number
*Bacillus cereus*	Bc	University of Queensland	UoQ 446
*Corynebacterium xerosis*	Cx	University of Melbourne	UoM 187
*Escherichia coli*	Ec	University of Queensland	UoM 182
*Proteus mirabilis*	Pm	University of Queensland	UoQ 21
*Proteus vulgaris*	Pv	University of Queensland	UoQ 22
*Pseudomonas aeruginosa*	Pa	University of Queensland	UoQ 16
*Shigella sonnei*	Shig	University of Queensland	UoQ 158
*Staphylococcus aureus*	Sa	University of Queensland	UoQ 111
*Staphylococcus epidermidis*	Se	University of Queensland	UoQ 105
*Streptococcus pyogenes*	Sp	La Trobe University	LTU 123

### Optimization of *Bovicola ovis* specific LAMP assay

Different primer concentrations and loop primer combinations were assessed using previously prepared standards during initial assay development, with LAMP reactions resulting in the fastest amplification times were carried out in 25 μL volumes using 15 μL OptiGene GspSSD 2.0 Isothermal Mastermix (ISO-DR004, OptiGene, Horsham, UK), 5 μL primer mix with final concentrations of 1.6 μm ea FIP and BIP, 0.2 μm ea F3 and B3 and 0.4 μm ea LF and LB (Table 2), and 5 μL template per manufacturer instructions. All runs were performed with a positive control and NTC reactions. Amplification was performed in a Genie II (OptiGene) real-time fluorometer, with an initial pre-heat of 40 °C—1 min, followed by amplification at 65 °C—30 min and anneal from 94 to 84 °C at 0.5 °C/s. Results were reported as time to positive (T_p_) in minutes and seconds (mm:ss), with T_p_s <20 min considered as positive amplification, and anneal derivative melting temperature (T_a_) reported in °C. Assay sensitivity and specificity was determined using the same bacterial panel and known negative fleece described previously.

## Results

### Analytical performance of *B. ovis* qPCR

**Fig. 1:**
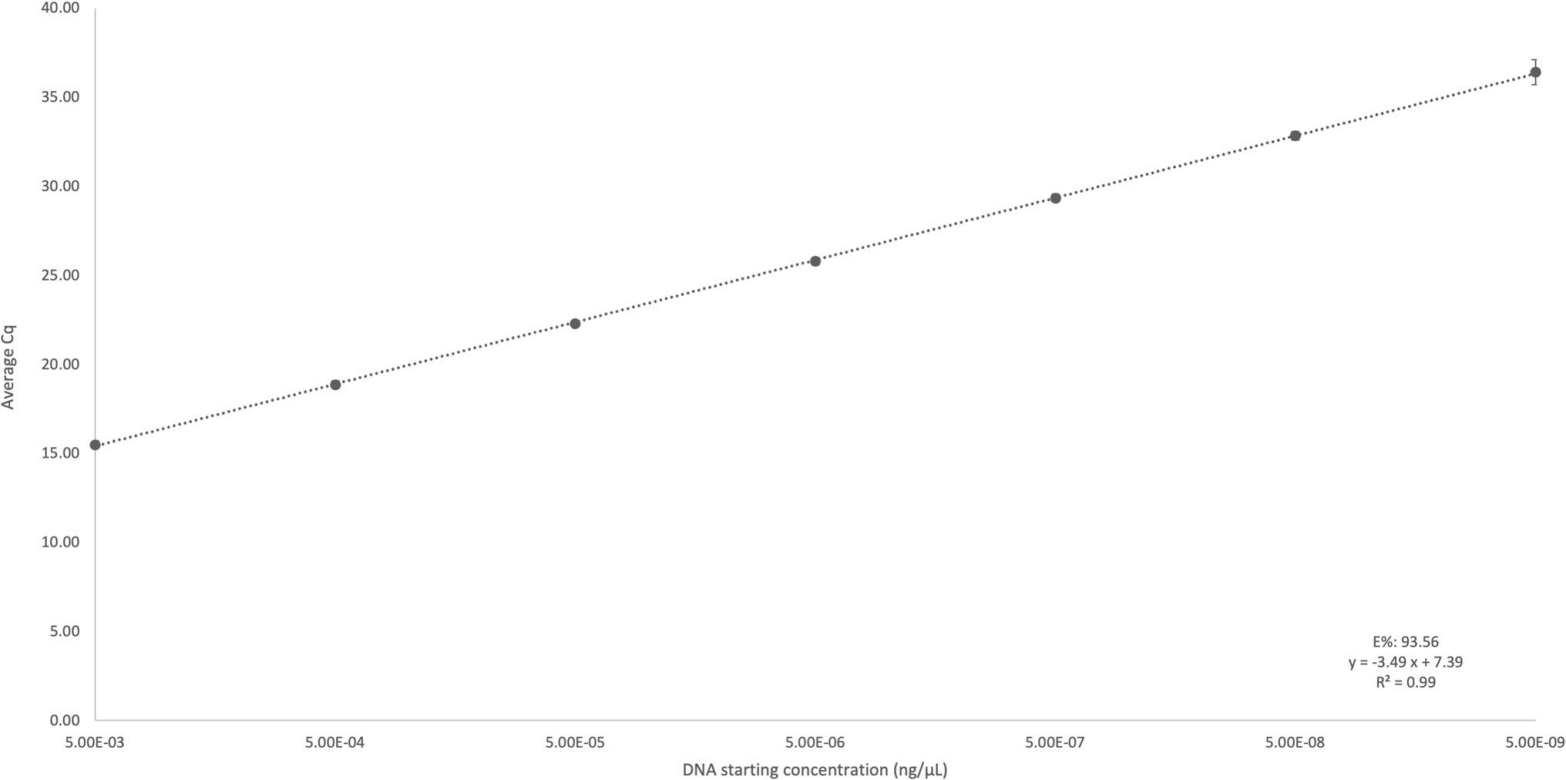
Standard curve for the *B. ovis* qPCR assay. A standard curve was generated by preparing a 10-fold serial dilution of *B. ovis* COI standards ranging from 5 × 10^−3^ to 5 × 10^−9^ ng/μL. This standard curve represents the mean Cq values performed over 10 runs. Error bars represent standard deviation

Assay sensitivity was evaluated using a standard curve using a serial dilution of *B. ovis* COI standards with starting concentrations of 5 × 10^−3^–5 × 10^−9^ ng/μL reliably amplifying 5 × 10^−8^ ng/μL with Cq ranges of 15–37. A standard curve representing the mean of ten replicate runs is shown in Fig. 1 providing a slope of −3.49*x* + 7.39, providing an average efficiency of 93.56%, and *R*^2^ of 0.99 (Fig. 1). 5 × 10^−8^ ng/μL was chosen as the limit of detection (LoD) of the assay as the standard curve was not linear from 5 × 10^−9^ ng/μL, with 0.7 Cq standard deviation. The standard deviation from that point was deemed unacceptable for reliable amplification (>0.5). However, the analytical sensitivity of 5 × 10^−8^ ng/μL reported here was sufficient, with the qPCR reliably amplifying *B. ovis* DNA from all positive fleece samples (Table 4).

**Table 4:** Average cycle quantification (Cq) values from the *B. ovis* qPCR of duplicate replicates from *B. ovis* negative and positive fleece

Negative fleece		Bacterial panel	
Sample	Average Cq	Sample	Average Cq
VIC03	–	*Bacillus cereus*	–
VIC04	–	*Corynebacterium xerosis*	–
VIC05	–	*Escherichia coli*	–
VIC06	–	*Proteus mirabilis*	–
VIC07	–	*Proteus vulgaris*	–
Positive fleece		*Pseudomonas aeruginosa*	–
VIC01	32.83	*Shigella sonnei*	–
VIC02	27.49	*Staphylococcus aureus*	–
SA01	36.85	*Staphylococcus epidermidis*	–
SA02	26.25	*Streptococcus pyogenes*	–
SA03	29.00	Neg extraction control	–

Assay analytical specificity was initially assessed using common bacterial species present in sheep fleece presented in Table 4. These samples failed to amplify DNA from the bacterial specificity panel (Table 4). Initially *Proteus vulgaris* had intermittent amplification, with one replicate returning a late Cq (Cq = 37.24). This sample was repeated and subsequently failed to amplify (Table 4). Assay sensitivity was further assessed using negative fleece previously presented in Table 1. Initially four of five fleece samples designated negative for *B. ovis* failed to amplify *B. ovis* DNA (Fig S2), with one sample, VIC04 amplifying with a late Cq (Cq = 36.20). This sample was re-run and subsequently failed to amplify consequently, VIC04 was considered negative for *B. ovis* DNA (Table 4, Fig S3). Considering that fleece samples are mixed samples with high levels of competing non-target DNA from various organisms, the lack of amplification from the bacterial specificity panel and from *B. ovis* negative fleece suggests that the *B. ovis* qPCR assay is highly specific and unlikely to amplify non-target species.

### Analytical performance of *B. ovis* LAMP

LAMP sensitivity was assessed using the same serial dilutions prepared for the qPCR standard curve, and used to determine the analytical sensitivity and to assess inter- and intra-assay variation of the *B. ovis* LAMP assay. Table 5 represents the mean of ten replicate runs, with samples performed in triplicate providing T_p_s between 5:45 and 29:15 in 30 min amplification time. The standard curve was unreliable from 5 × 10^−7^ ng/μL, with the coefficient of variation from that point deemed unacceptable (>10%). At a starting concentration of 5 × 10^−7^ ng/μL, T_p_s ranged from 10:45 to 29:15 between replicates, with frequent intermittent amplification between inter-assay replicates. Assay LoD was therefore determined to be 5 × 10^−6^ ng/μL as variation was <10%, with all replicates consistently amplifying (Table 5).

**Table 5:** Inter-assay coefficient of variation of *B. ovis* standards using LAMP

ng/μL	Average T_p_ (±SD)	Inter-assay CV (%)
5 × 10^−3^	06:13 ± 0.11 SD	1.83
5 × 10^−4^	06:97 ± 0.21 SD	2.95
5 × 10^−5^	07:76 ± 0.34 SD	4.36
5 × 10^−6^	09:37 ± 0.51 SD	5.46
5 × 10^−7^	13:04 ± 1.99 SD	15.28

*SD*, standard deviation; *CV*, coefficients of variability

Additionally, the *B. ovis* LAMP demonstrated high analytical specificity with no non-target amplification of the bacterial specificity panel used during qPCR validation, or known negative fleece samples (Supplementary Table 2; Fig S4). Although the LoD for the *B. ovis* LAMP is 100-fold less sensitive in comparison to the *B. ovis* qPCR reported as 5 × 10^−8^ ng/μL, the *B. ovis* LAMP could still reliably detect all lice positive fleece samples, with T_p_ values ranging between 8 and 15 min (Table 6, Fig S5). The high sensitivity of the *B. ovis* LAMP is further supported with samples used in this study providing T_p_s from lice positive fleece in less than 15 min using the dissolved fleece method despite these samples containing high levels of mixed DNA.

**Table 6:** Average amplification times from the *B. ovis* LAMP of duplicate replicates from *B. ovis* positive lice samples

Sample code	Average T_p_ (±SD)
VIC01	09:15 (±0)
VIC02	13:80 (±2.12)
SA01	07:30 (±0.21)
SA02	07:80 (±0.49)
SA03	08:30 (±0)

*T*_*p*_, time to positive; *SD*, standard deviation

### Comparison of *B. ovis* qPCR and LAMP performance

Lice detection from dissolved fleece samples was assessed using three methods. Samples were initially assessed by eye during the weighing process to confirm the presence or absence of lice. These samples were scored as positive or negative for *B. ovis* based on physical louse detection. As there is currently no test considered as gold standard for *B. ovis* detection, visual assessment was initially designated the reference assay through the development of the *B. ovis* qPCR, with the qPCR subsequently designated as the reference assay during LAMP development. All samples in this study were analyzed using both *B. ovis* molecular assays. All negative fleece (*n* = 5/5) failed to amplify using both detection methods, with all positive fleece (*n* = 5/5) successfully amplifying *B. ovis* DNA with both qPCR and LAMP methods (Table 7). Between all three methods used, each method had 100% assay specificity irrespective of the assessment method used. Both molecular methods presented here only amplified lice positive samples with no non-target amplification observed in negative fleece samples.

**Table 7:** Comparison of all detection methods used for *B. ovis* identification in this study

Sample code	Visual assessment	Average Cq	Average T_p_
		qPCR	LAMP
VIC01	+	32.83	09:15
VIC02	+	27.49	13:80
VIC03	−	–	–
VIC04	−	–	–
VIC05	−	–	–
VIC06	−	–	–
VIC07	−	–	–
SA01	+	36.85	07:30
SA02	+	26.25	07:80
SA03	+	29.00	08:30

*T*_*p*_, time to positive

### Assessing field-appropriate sampling methods

Given all lice positive samples successfully amplified *B. ovis* DNA on qPCR and LAMP, fleece swabbing was assessed to see if this method was a suitable method for in-field processing using minimal equipment and processing methods. Average processing times per sample from initial sample preparation to LAMP reaction set-up was less than 20 min, drastically reducing sample preparation time. The swabbing method when compared to dissolved fleece samples resulted in 100% detection of positive fleece samples, with the *B. ovis* LAMP reporting positive T_p_s from all lice positive samples ranging from 10:30–18:00 min (Table 8, Fig S6). The initial failure of SA02 to amplify despite being qPCR positive may be attributed to the carry-over presence of inhibitory substances, such as dirt, or possibly the Chelex resin being unintentionally transferred into the LAMP reaction. This sample was re-run and subsequently returned an average positive T_p_ of 17:58 (Fig S6). Though these T_p_s are up to 77% slower compared to dissolved fleece samples, swabbing greatly improved sample throughput, with the reduction in T_p_ values not affecting the overall LAMP run time. Using the swabbing method which removed the requirement for dedicated laboratory equipment needed for dissolving fleece, ten samples were prepared in less than 20 min in contrast to dissolved fleece samples which require a minimum 2 h incubation at −80 °C for DNA precipitation.

**Table 8:** Comparison of *B. ovis* LAMP amplification times using dissolved fleece and swabbing methods. Detection rate was determined by both duplicate replicates returning positive T_p_ values across both sampling methods

Sample code	Average T_p_ and (±SD) values		% difference
	Dissolved fleece	Swabbed fleece	
VIC01	09:15 (±0)	16:73 (±1.80)	58.58
VIC02	13:80 (±2.12)	16:32 (±0.11)	16.73
SA01	07:30 (±0.21)	13:88 (±0.60)	62.13
SA02	07:80 (±0.49 )	17:58 (±0.60)	77.07
SA03	08:30 (±0)	10:65 (±0.49)	24.8
Detection rate	100%		

*T*_*p*_, time to positive; *SD*, standard deviation

## Discussion

Sheep lice infestations are an important cause of production losses in dual purpose sheep flocks (Lane et al. 2015; McLeod 1995). Despite this, the prevalence of *B. ovis* in Australia is poorly understood largely due to issues with available testing methods (Popp et al. 2012). It is currently expected that producers individually assess each suspected infected animal using visual assessment to confirm infection. Further, reliance on insecticides to treat infestations may reduce (but not eliminate) infestation in sheep, and this can result in false negative using visual inspection (Horton and Carew 2014). Though visual assessment does not require dedicated equipment, it is time consuming, laborious, and has limited use in situations involving sale yards, stray sheep, and during shearing where processing time is critical.

Previous attempts to develop a diagnostic tool to replace visual detection methods resulted in a commercial ELISA for *B. ovis* detection (Wojtek et al. 2001). The commercial ELISA required a minimum of 3 days to obtain results and lack of adoption by producers was attributed to long processing times and high cost (AUD $134 per sample), with recent attempts to improve current detection methods for *B. ovis* including the development of a PCR and LAMP assay by Wong et al. (2020).

This study reported a limit of detection of 5.6 × 10^−4^ and 5.6 × 10^−3^ ng/μL for PCR and LAMP, respectively, using a crude boiling method from shearing combs, with use of a commercial DNA extraction kit resulting in sensitivity declining 10-fold with detection limits of 5.6 × 10^−3^ and 5.6 × 10^−2^ ng/μL for PCR and LAMP, with both NAATs requiring at least 1 h amplification time and additional sample preparation time (Wong et al. 2020). Reduced specificity was also reported with false positive *B. ovis* amplification using the rapid boiling method which assay specificity declining from 90–91.7% to 33% for PCR and 75% for LAMP against the studies gold standard method of PCR using samples prepared from a commercial DNA extraction kit (Wong et al. 2020). The authors concluded that false positive amplification from boiled samples was likely the result of non-specific amplification, but their study did not sequence these amplicons to determine what was being amplified. Despite these shortcomings, this study provides some improvements to visual detection through increased sensitivity and reduced reliance on visual detection, but is still limited by pooled sample testing which is incapable of differentiating individual infestations.

Individual sampling is of greater benefit to pooled sampling in the contexts of stray sheep management through the incursion of potentially lousy sheep from neighboring properties; the return of stray sheep that may have had contact with a lousy mob; purchasing or selling potentially lousy sheep at sale yards; and routine animal checks where wool rubbing is observed and infestation is suspected. In these situations, clipper sample submission is not feasible; hence, individual fleece clippings or a swab would be a convenient alternative. This study describes two reliable, rapid, and cost-effective diagnostic tools which can detect *B. ovis* in both the laboratory and field setting. Two key advantages of the qPCR and LAMP described are shorter time to results and flexibility of timing with sample collection. The qPCR and LAMP assays described in the present study offer several advantages over the commercial ELISA and previous work by Wong et al. (2020) in terms of improved analytical performance, reduced time to results and increased flexibility because collection of wool samples can be conducted at any time, and is not restricted to shearing time. Additionally, the methods presented in this study do not require physically finding *B. ovis* (Horton and Carew 2014). Although the methods reported in this study are from individually sampled sheep, they have the potential to be adapted for pooled sampling approaches for *B. ovis* detection.

Both assays described in this study show high specificity (100%) for non-target DNA amplification, failing to amplify DNA from lice negative fleece contaminated with various microorganisms (Lyness et al. 1994) in addition to the pure bacterial genomic DNA used, while accurately amplifying *B. ovis* target DNA from all *B. ovis* positive dissolved fleece, with high sensitivity (5 × 10^−8^ and 5 × 10^−6^ ng/μL for qPCR and LAMP, respectively). These results suggest that the *B. ovis* qPCR and LAMP assays can detect low levels of lice DNA, critical for detecting low level infestations particularly after shearing when lice numbers are heavily reduced, but have not been fully eradicated (Lucas et al. 2017b).

Although the *B. ovis* qPCR presented here focuses on disease management, this method can be adapted for routine disease surveillance through the submission of wool samples for other sheep ectoparasites including mites, keds, and ticks which are notoriously difficult to detect (Taylor 2012). As the *B. ovis* qPCR presented in this study multiplexes for both sheep lice and sheep DNA, it could be modified to add additional targets as required, further improving diagnostic capacity. Modifying the *B. ovis* qPCR for surveillance studies would greatly benefit livestock health through enabling disease monitoring, particularly as wild animals can potentially spread pathogens to other livestock and humans (Huaman et al. 2020).

The development of the qPCR and LAMP assay using dissolved wool provided consistent amplification for both assays, with minimal intra-assay variation observed between replicates. Low variability between the replicates suggested that there was negligible DNA inhibition for qPCR and LAMP despite fleece containing numerous organic contaminants known to interfere with DNA amplification such as humic substances. It is possible that dilution of samples 1/10 prior to amplification reduced potential inhibitors and therefore the likelihood of failed amplification (Alaeddini 2012; Monteiro et al. 1997; Schrader et al. 2012). Although diluting samples reduced available DNA quantity, the methods described in this present study reliably amplified *B. ovis* DNA and are substantially faster than the ELISA. However, processing times could be further reduced for field-appropriate sampling as dissolving wool requires a minimum 2 h incubation period solely for sample preparation. As such, fleece swabbing was assessed and used in conjunction with the LAMP assay. Although there are several benefits to fleece sampling, some consideration to sampling approaches is required owing to sheep lice biology. It is recommended that sampling is prioritized on shearing status, with unshorn sheep sampled from the sides and back within 6 mm from the skin surface, with shorn sheep requiring greater attention to the neck and lower body regions where wool is generally left longer. As samples were submitted to us from producers, we were unable to assess the effects of sampling methodology on assay performance.

Regardless, swabbing resulted in T_p_s for all lice positive samples within 20 min, with total sample preparation time between 15 and 20 min and a cumulative time to result of less than 1 h using this workflow. This suggests that the LAMP assay can be adapted for in-field use as a point of care test (POCT), with minimal processing and handling steps subsequently providing results within an hour without leaving the paddock. Further assessment of the *B. ovis* LAMP and swabbing method described here could see this workflow being deployed as a POCT for field use during routine animal health inspections, or at sale yards without requiring central laboratory access Furthermore the LAMP method could be modified for point of care detection of other important pathogens of sheep such as the differentiation of virulent and benign footrot demonstrated by Best et al. (2018) in-field.

## Conclusion

This study reports the development of a qPCR and LAMP assay to detect *B. ovis* DNA in sheep wool samples. To the best of our knowledge, this is the first description of a multiplex qPCR assay for the simultaneous detection of *B. ovis* and *O. aries* in fleece samples. The *B. ovis* LAMP method has potential for point of care testing with acceptable sensitivity, specificity, and faster sample throughput using swabbed wool samples. Improved detection of *B. ovis* in conjunction with appropriate lice management and surveillance strategies can reduce the economic impacts of *B. ovis* in Australian sheep.

## Supplementary Information

ESM 1 (DOCX 16 kb)

Fig. S1 (PNG 1164 kb)

High Resolution (TIFF 14826 kb)

Fig. S2 (PNG 1201 kb)

High Resolution (TIFF 14826 kb)

Fig. S3 (PNG 448 kb)

High Resolution (TIFF 14826 kb)

Fig. S4 (PNG 646 kb)

High Resolution (TIFF 17686 kb)

Fig. S5 (PNG 625 kb)

High Resolution (TIFF 14826 kb)

Fig. S6 (PNG 1150 kb)

High Resolution (TIFF 14826 kb)

## References

[CR1] Alaeddini R (2012). Forensic implications of PCR inhibition—a review. Forensic Sci Int Genet.

[CR2] Amoah ID, Singh G, Stenström TA, Reddy P (2017). Detection and quantification of soil-transmitted helminths in environmental samples: a review of current state-of-the-art and future perspectives. Acta Trop.

[CR3] Barkway CP, Pocock RL, Vrba V, Blake DP (2011). Loop-mediated isothermal amplification (LAMP) assays for the species-specific detection of *Eimeria* that infect chickens. BMC Vet Res.

[CR4] Bath C et al (2020) Further development of a reverse-transcription loop-mediated isothermal amplification (RT-LAMP) assay for the detection of foot-and-mouth disease virus and validation in the field with use of an internal positive control. Transbound Emerg Dis n/a(n/a). 10.1111/tbed.13589 10.1111/tbed.13589 32311239

[CR5] Best N, Rodoni B, Rawlin G, Beddoe T (2018). The development and deployment of a field-based loop mediated isothermal amplification assay for virulent *Dichelobacter nodosus* detection on Australian sheep. PLoS One.

[CR6] Czeglédi I (2021). Congruency between two traditional and eDNA-based sampling methods in characterising taxonomic and trait-based structure of fish communities and community-environment relationships in lentic environment. Ecol Indic.

[CR7] Djurhuus A et al (2017) Evaluation of filtration and DNA extraction methods for environmental DNA biodiversity assessments across multiple trophic levels. Front Mar Sci 4(314). 10.3389/fmars.2017.00314

[CR8] Gandasegui J, Fernández-Soto P, Hernández-Goenaga J, López-Abán J, Vicente B, Muro A (2016). Biompha-LAMP: a new rapid loop-mediated isothermal amplification assay for detecting *Schistosoma mansoni* in *Biomphalaria glabrata* snail host. PLoS Negl Trop Dis.

[CR9] Hoffmann B (2009). A review of RT-PCR technologies used in veterinary virology and disease control: sensitive and specific diagnosis of five livestock diseases notifiable to the World Organisation for Animal Health. Vet Microbiol.

[CR10] Horton BJ, Carew AL (2014). A comparison of deterministic and stochastic models for predicting the impacts of different sheep body lice *Bovicola ovis* management practices. Anim Prod Sci.

[CR11] Horton BJ, Bailey A, Carew AL (2015). A regional model of sheep lice management practices for predicting the impact of treatment for lice when no lice are detected. Anim Prod Sci.

[CR12] Huaman JL (2020). Serosurveillance and molecular investigation of wild deer in Australia reveals seroprevalence of Pestivirus infection. Viruses.

[CR13] James PJ (2010). Issues and advances in the integrated control of sheep lice. Anim Prod Sci.

[CR14] Lane J, Jubb T, Shephard R, Webb-Ware J, Fordyce G (2015) Priority list of endemic diseases for the red meat industries (Final Report No B. AHE. 0020).

[CR15] Li J-F, Sheen J, Sucher NJ, Hennell JR, Carles MC (2012). DNA purification from multiple sources in plant research with homemade silica resins. Plant DNA fingerprinting and barcoding: methods and protocols.

[CR16] Lucas PG, Horton BJ, Parsons D, Carew AL (2017). A regional model of sheep lice management practices to examine the impact of managing straying sheep combined with other management choices. Anim Prod Sci.

[CR17] Lucas PG, Horton BJ, Parsons D, Carew AL (2017). A regional model of sheep lice to study the effect on lice prevalence and costs for Australian farms using a range of treatment efficacy in combination with other lice control strategies. Anim Prod Sci.

[CR18] Lyness EW, Pinnock DE, Cooper DJ (1994). Microbial ecology of sheep fleece. Agric Ecosyst Environ.

[CR19] McLeod RS (1995). Costs of major parasites to the Australian livestock industries. Int J Parasitol.

[CR20] Monteiro L (1997). Complex polysaccharides as PCR inhibitors in feces: *Helicobacter pylori* model. J Clin Microbiol.

[CR21] Morcombe P, Young G, Ball M, Dunlop R (1996). The detection of lice (*Bovicola ovis*) in mobs of sheep: a comparison of fleece parting, the lamp test and the table locks test. Aust Vet J.

[CR22] Nagamine K, Hase T, Notomi T (2002). Accelerated reaction by loop-mediated isothermal amplification using loop primers. Mol Cell Probes.

[CR23] Niven D, Pritchard D (1985). Effects of control of the sheep body louse *Damalinia ovis* on wool production and quality. Aust J Exp Agric.

[CR24] Noden BH, Martin J, Carrillo Y, Talley JL, Ochoa-Corona FM (2018). Development of a loop-mediated isothermal amplification (LAMP) assay for rapid screening of ticks and fleas for spotted fever group rickettsia. PLoS One.

[CR25] Notomi T et al (2000) Loop-mediated isothermal amplification of DNA. Nucleic Acids Res 28. 10.1093/nar/28.12.e63 10.1093/nar/28.12.e63 PMC102748 10871386

[CR26] Parker AM, Mohler VL, Gunn AA, House JK (2020). Development of a qPCR for the detection and quantification of *Salmonella* spp. in sheep feces and tissues. J Vet Diagn Investig.

[CR27] Poole CB (2017). Colorimetric tests for diagnosis of filarial infection and vector surveillance using non-instrumented nucleic acid loop-mediated isothermal amplification (NINA-LAMP). PLoS One.

[CR28] Popp S, Eppleston J, Watt BR, Mansfield S, Bush RD (2012). The prevalence of lice (*Bovicola ovis*) in sheep flocks on the central and southern Tablelands of New South Wales. Anim Prod Sci.

[CR29] Rathinasamy V (2018). Development of a multiplex quantitative PCR assay for detection and quantification of DNA from *Fasciola hepatica* and the intermediate snail host, *Austropeplea tomentosa*, in water samples. Vet Parasitol.

[CR30] Schrader C, Schielke A, Ellerbroek L, Johne R (2012). PCR inhibitors – occurrence, properties and removal. J Appl Microbiol.

[CR31] Taylor MA (2012). Emerging parasitic diseases of sheep. Vet Parasitol.

[CR32] Wang RF, Cao WW, Cerniglia CE (1997). A universal protocol for PCR detection of 13 species of foodborne pathogens in foods. J Appl Microbiol.

[CR33] Wojtek P, Michalski PY, Brian Shiell, Garry Levot (2001) Development of a lice detection test for “on-farm” use. Proceedings of the FLICS Conference

[CR34] Wong SA, Woodgate RG, Pant SD, Ghorashi SA (2020). Rapid detection of *Bovicola ovis* using colourimetric loop-mediated isothermal amplification (LAMP): a potential tool for the detection of sheep lice infestation on farm. Parasitol Res.

[CR35] Yang S, Rothman RE (2004). PCR-based diagnostics for infectious diseases: uses, limitations, and future applications in acute-care settings. Lancet Infect Dis.

