# Supplementary material for: Development of molecular detection methods of Bovicola ovis from sheep fleece
Source: Parasitol Res. 2022 Apr 18;121(6):1597–606. doi: 10.1007/s00436-022-07520-9 (PMC9098604; doi:10.1007/s00436-022-07520-9)
Supplement: Supplementary file 1 — (DOCX 16 kb) [file 436_2022_7520_MOESM1_ESM.docx]

**Supplementary Table 1. Quantitative PCR primers used in multiplex detection of B. ovis and O. aries**

| Target | Primer name | Sequence 5’-3’ | Amplicon size (bp) | Nucleotide position |
| --- | --- | --- | --- | --- |
| *B. ovis* | qBov3_F | TGTCTTGGTCCGTTTCTGGG | 108 | 1058 |
|  | qBov3_Pr | **FAM**-ACTTTGGGTGGTTTAACAGGCTTGG-**BHQ1** |  | 1108 |
|  | qBov3_R | GGAGAACCACGTCCACACAA |  | 1146 |
| *O. aries* | CB_Ovis_F | GGCACAAACCTAGTCGAATGAATC | 109 | 445 |

**Supplementary Table 2. Average amplification values from the B. ovis LAMP of duplicate replicates from the bacterial specificity panel. No positive amplification was observed.**

| Sample | Average T_P_ |
| --- | --- |
| Bacterial specificity panel |  |
| *Bacillus cereus* | - |
| *Corynebacterium xerosis* | - |
| *Eschericia coli* | - |
| *Proteus mirabilis* | - |
| *Proteus vulgaris* | - |
| *Pseudomonas aeruginosa* | - |
| *Shigella sonnei* | - |
| *Staphylococcus aureus* | - |
| *Staphylococcus epidermidis* | - |
| *Strepcococcus pyogenes* | - |
| Negative fleece |  |
| VIC03 | - |
| VIC04 | - |
| VIC05 | - |
| VIC06 | - |
| VIC07 | - |

T_p_ – Time to positive
