# Supplementary figures and images for: Development of molecular detection methods of Bovicola ovis from sheep fleece
Source: Parasitol Res. 2022 Apr 18;121(6):1597–606. doi: 10.1007/s00436-022-07520-9 (PMC9098604; doi:10.1007/s00436-022-07520-9)

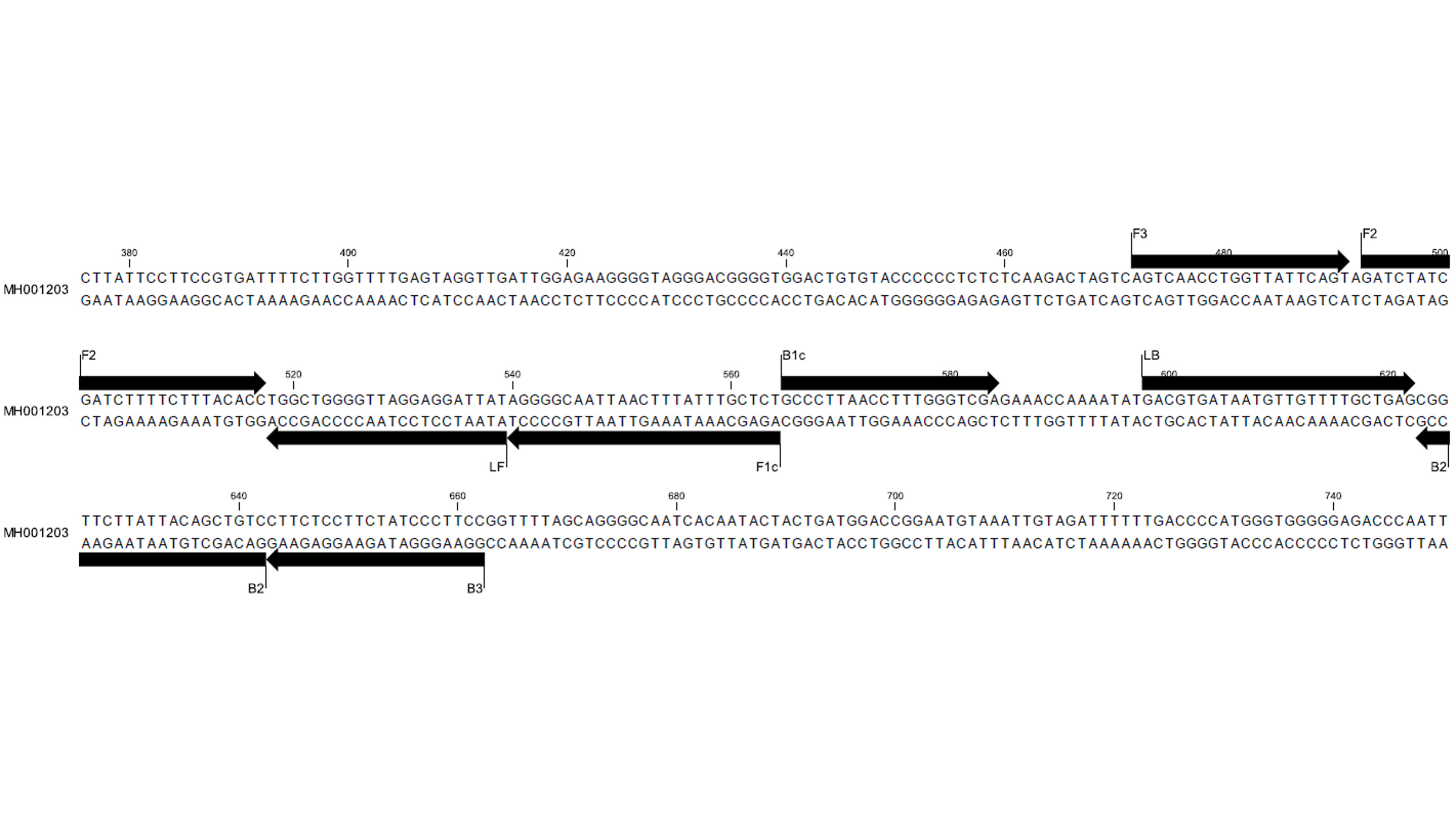

Supplement: Supplementary file 2 — (PNG 1164 kb) [file 436_2022_7520_Fig2_ESM.png]

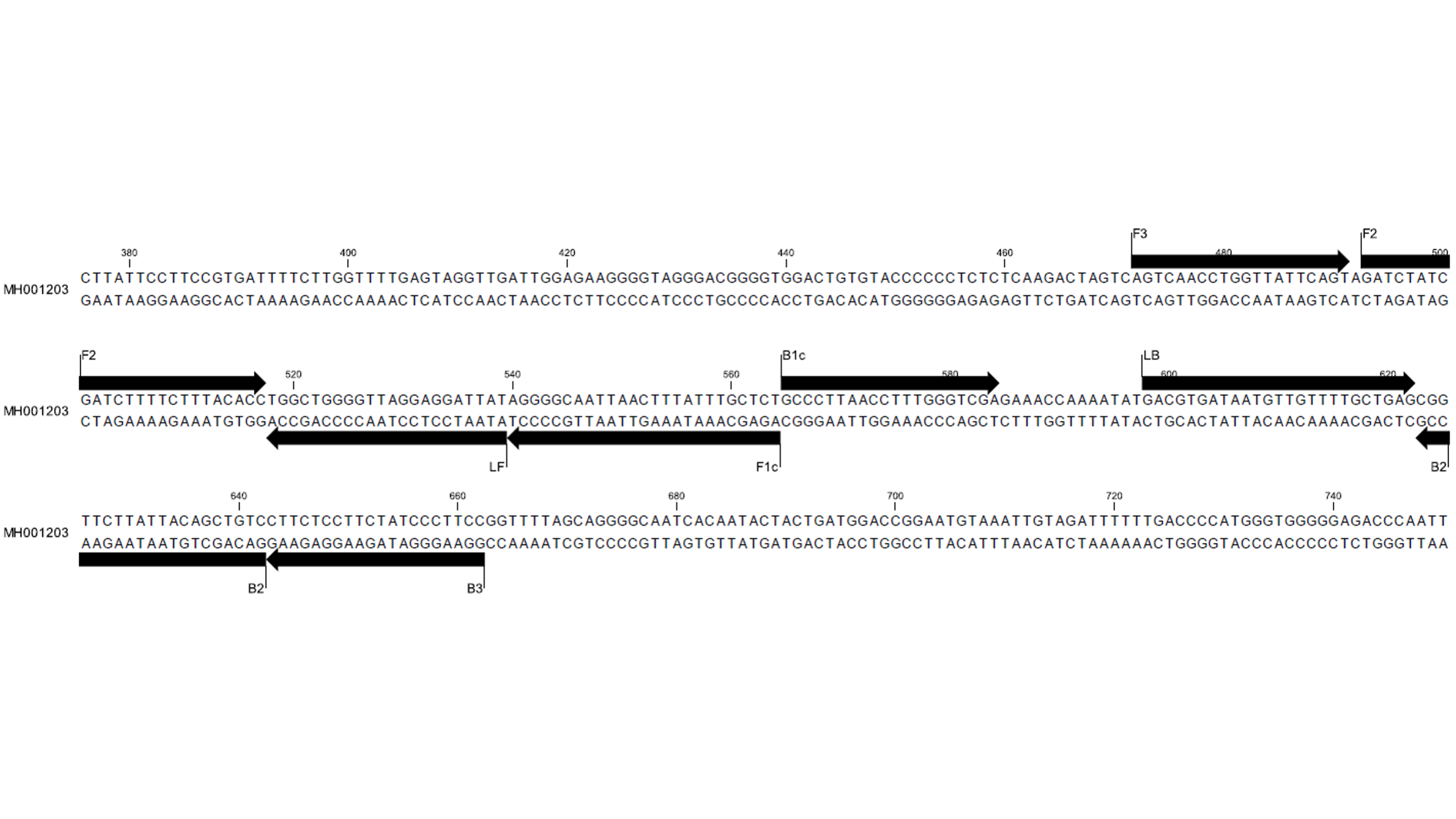

Supplement: Supplementary file 3 — High Resolution (TIFF 14826 kb) [file 436_2022_7520_MOESM2_ESM.tiff]

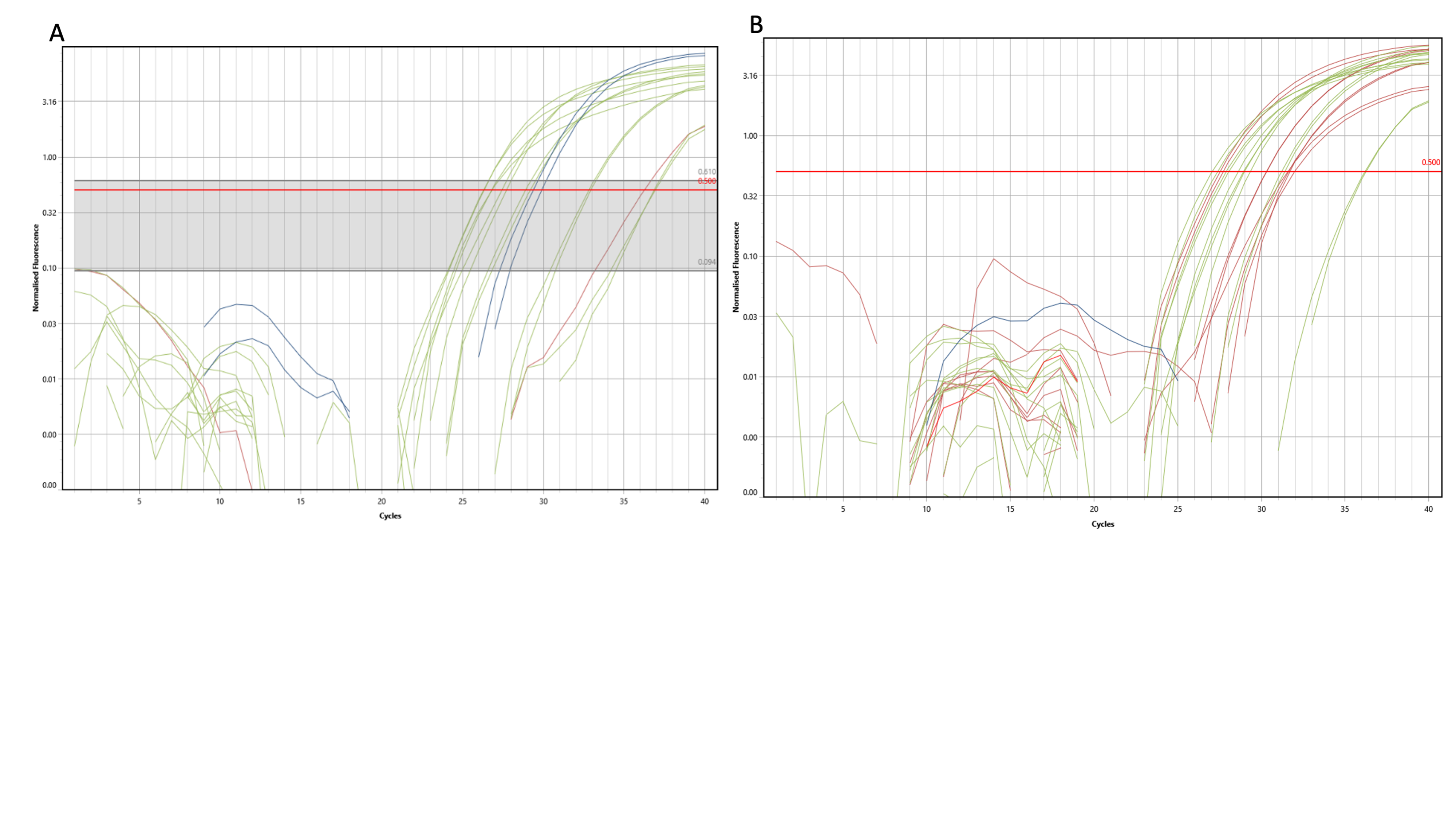

Supplement: Supplementary file 4 — (PNG 1201 kb) [file 436_2022_7520_Fig3_ESM.png]

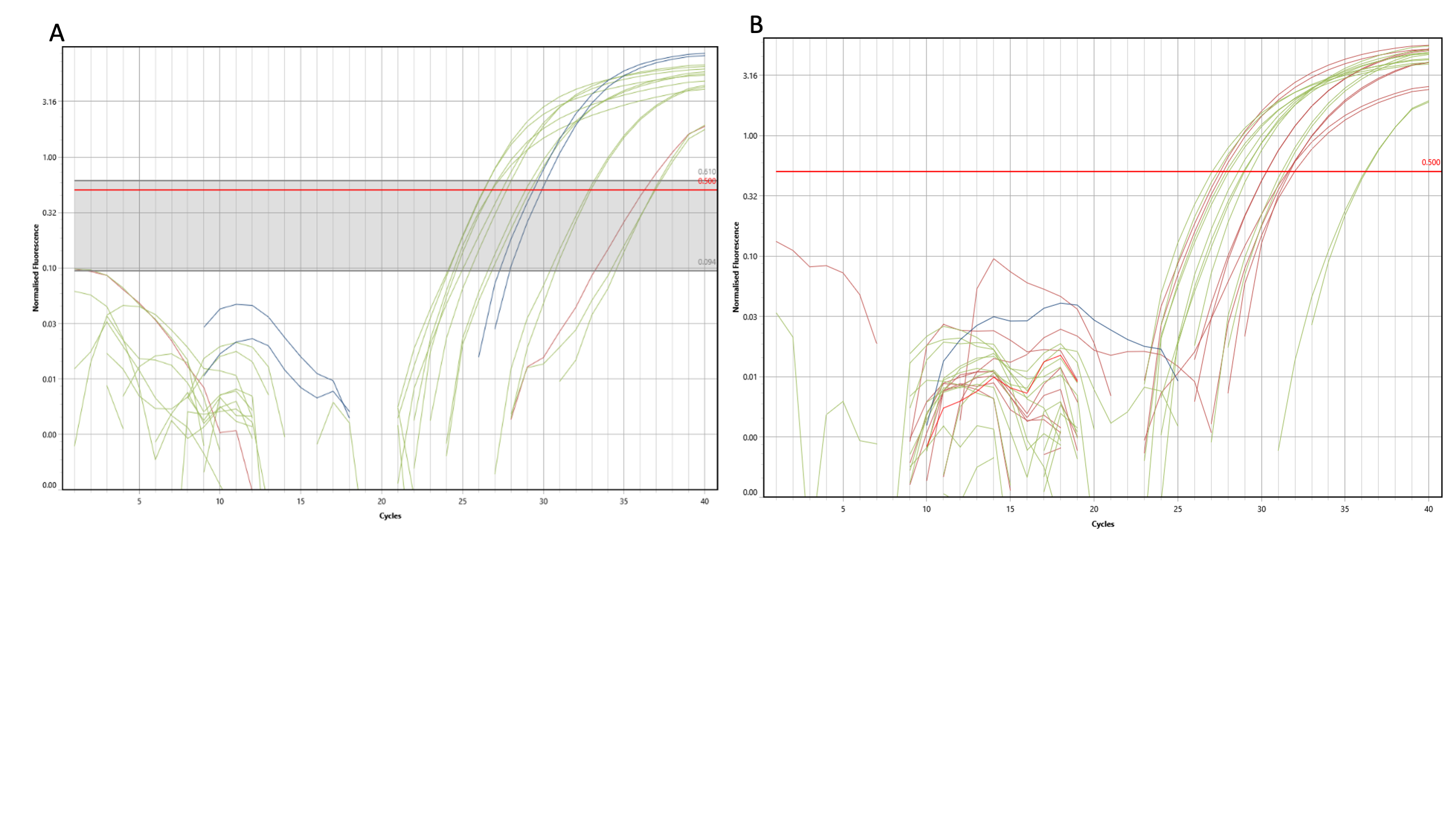

Supplement: Supplementary file 5 — High Resolution (TIFF 14826 kb) [file 436_2022_7520_MOESM3_ESM.tiff]

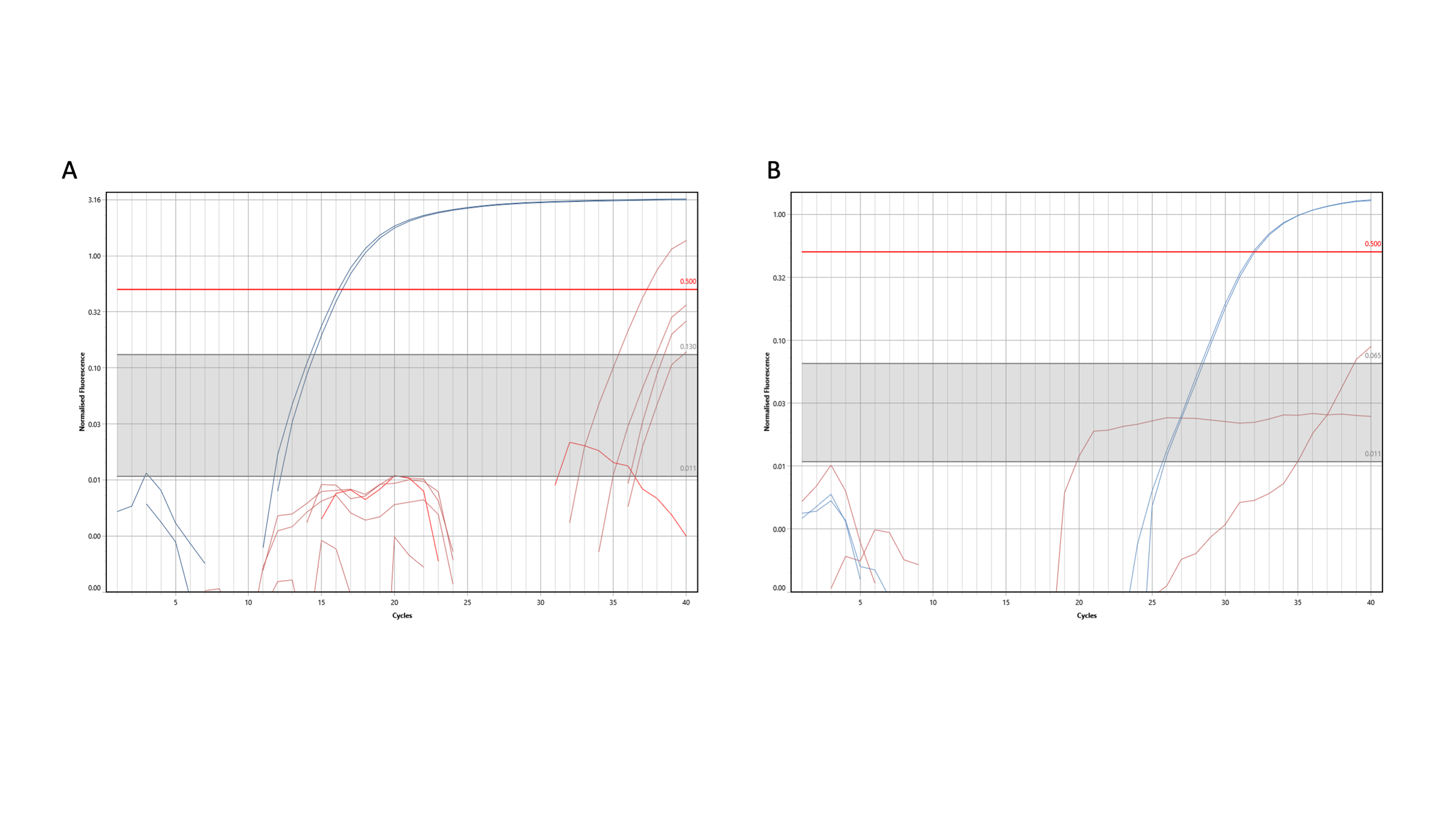

Supplement: Supplementary file 6 — (PNG 448 kb) [file 436_2022_7520_Fig4_ESM.png]

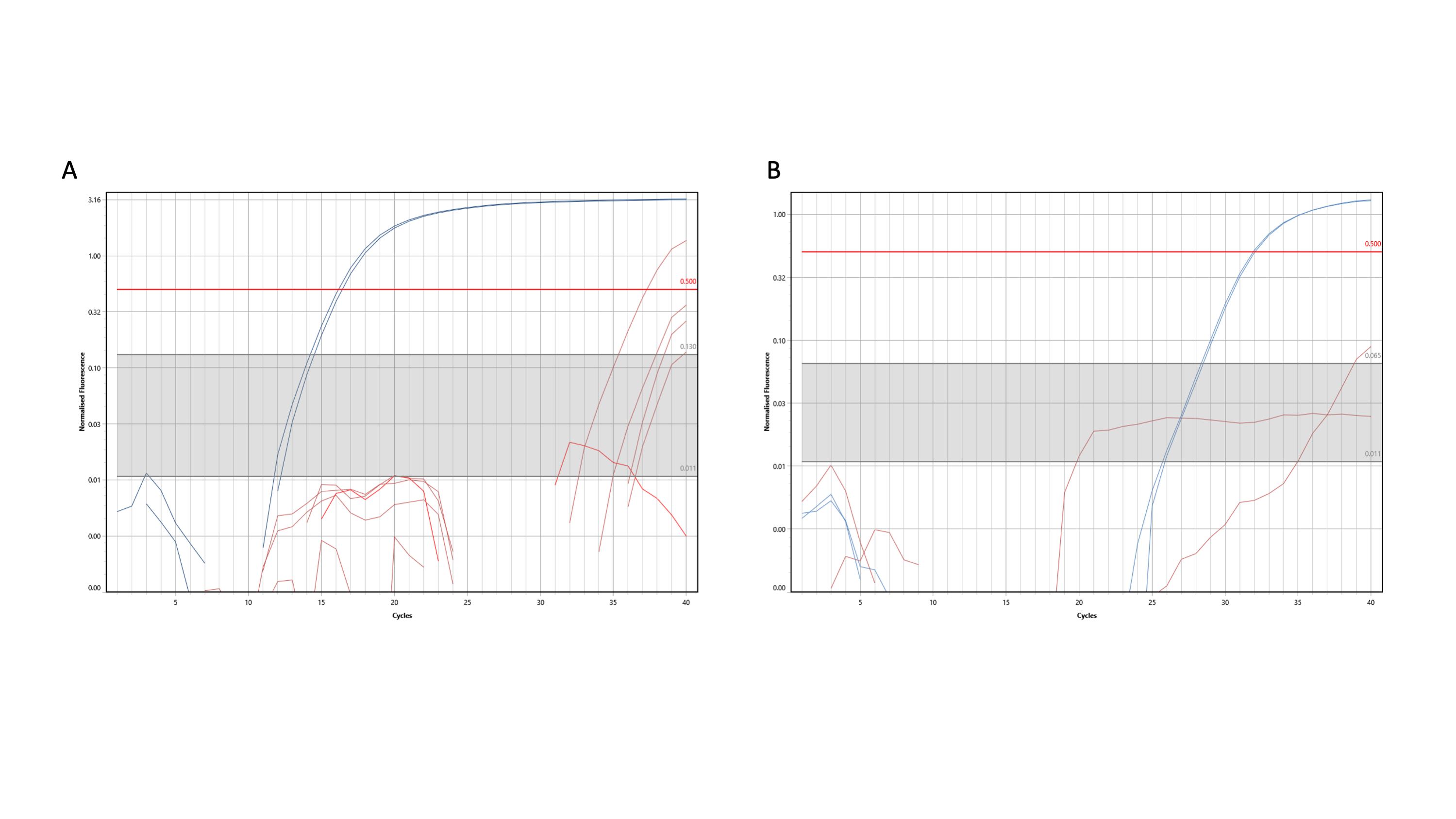

Supplement: Supplementary file 7 — High Resolution (TIFF 14826 kb) [file 436_2022_7520_MOESM4_ESM.tiff]

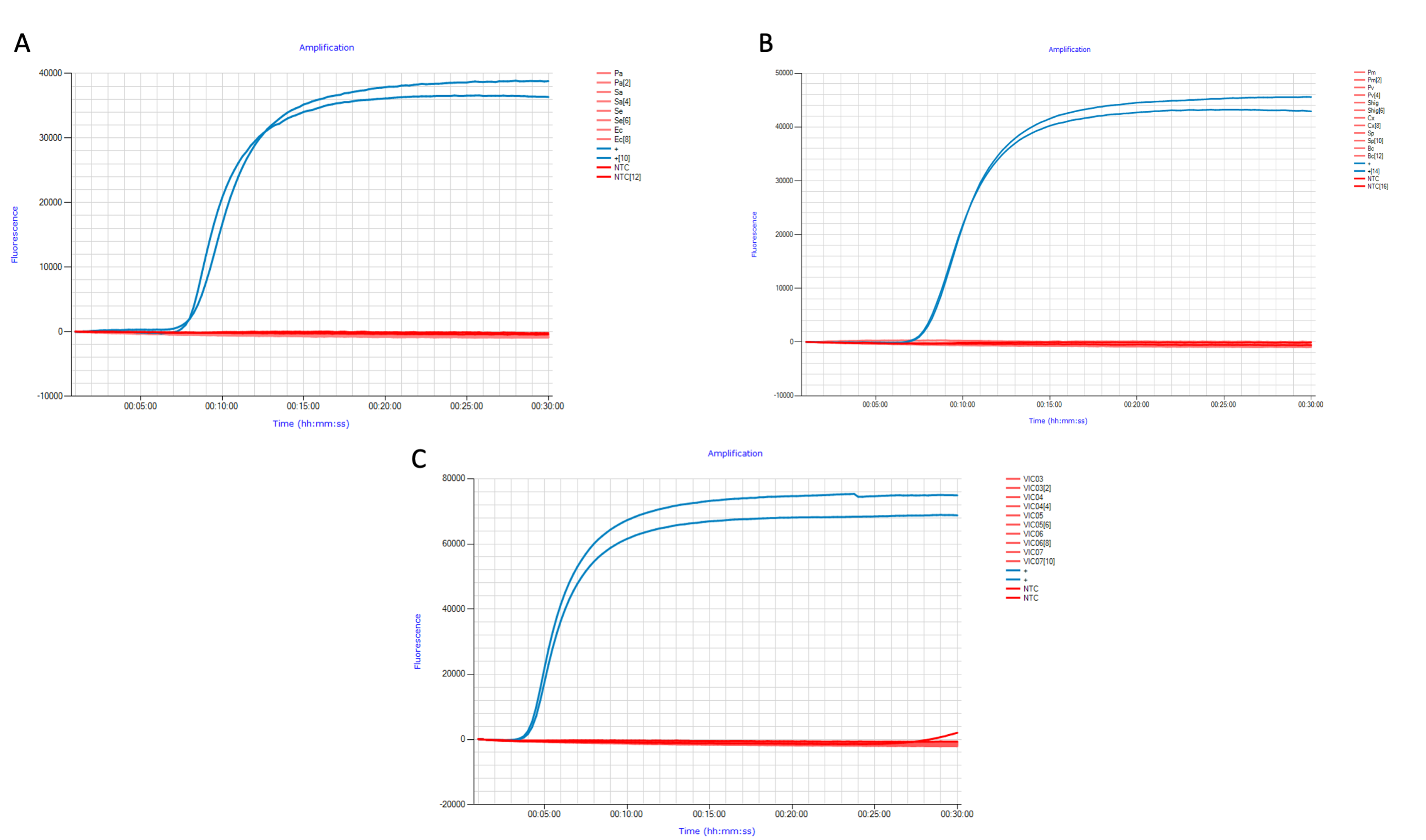

Supplement: Supplementary file 8 — (PNG 646 kb) [file 436_2022_7520_Fig5_ESM.png]

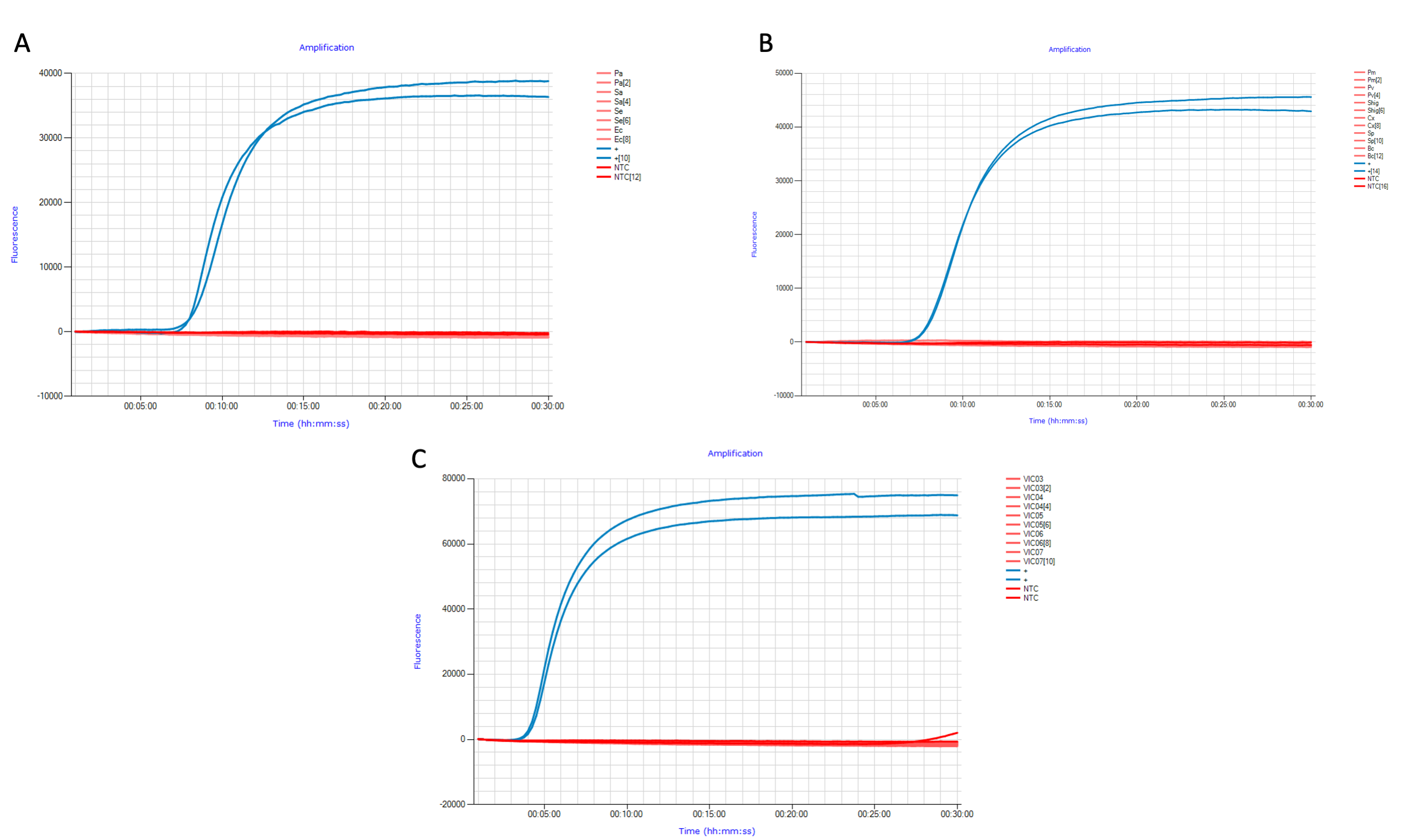

Supplement: Supplementary file 9 — High Resolution (TIFF 17686 kb) [file 436_2022_7520_MOESM5_ESM.tiff]

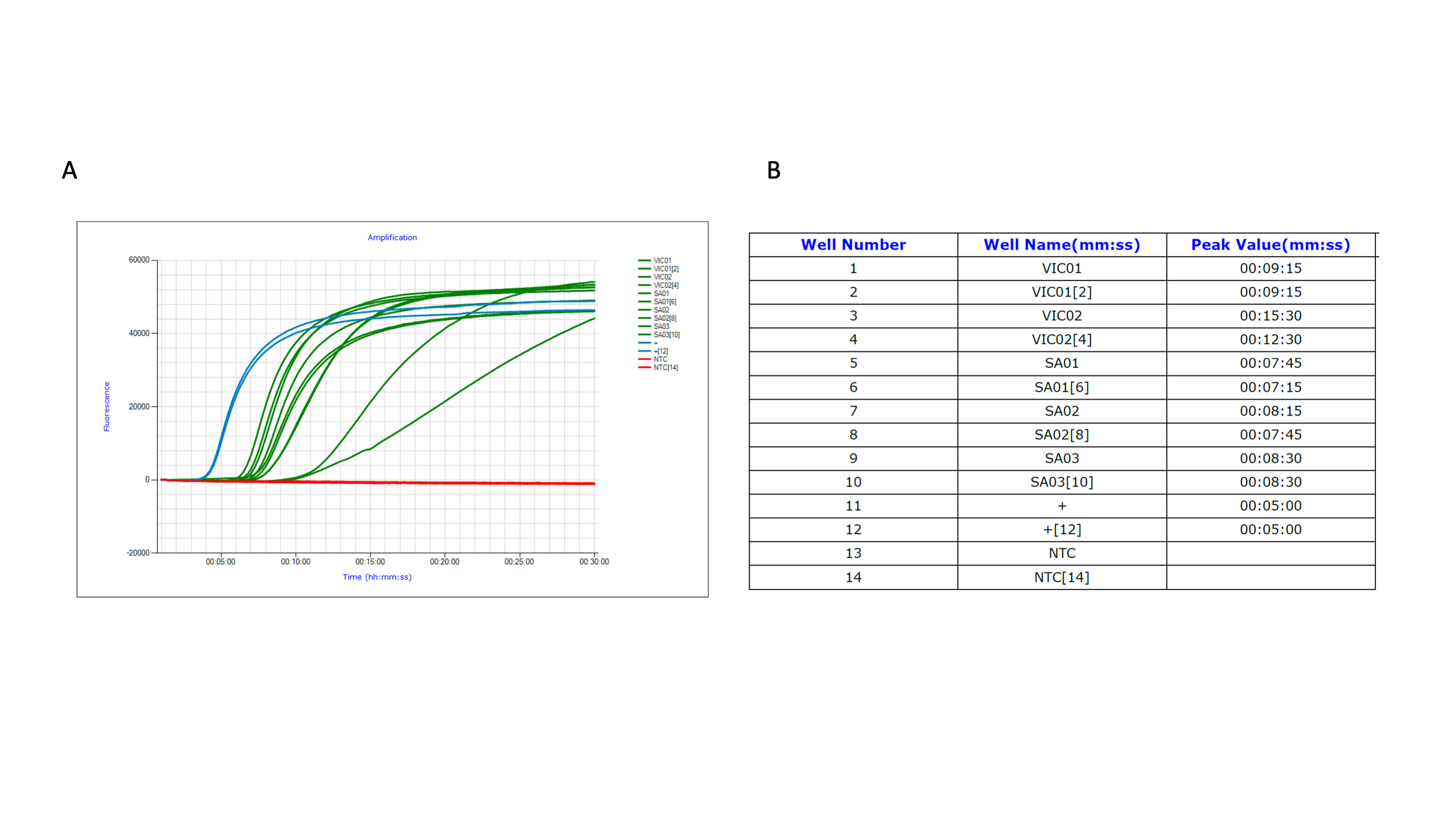

Supplement: Supplementary file 10 — (PNG 625 kb) [file 436_2022_7520_Fig6_ESM.png]

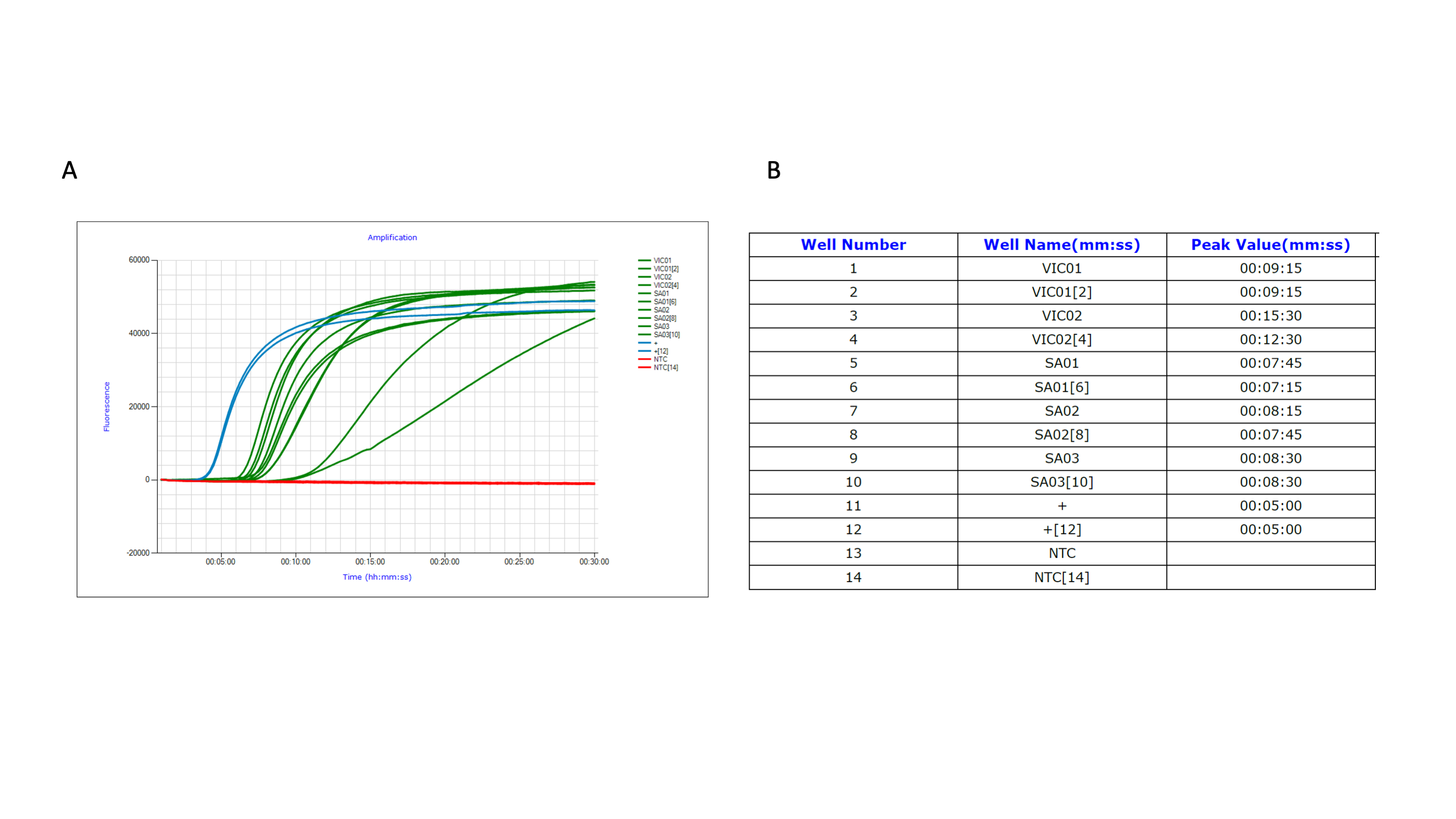

Supplement: Supplementary file 11 — High Resolution (TIFF 14826 kb) [file 436_2022_7520_MOESM6_ESM.tiff]

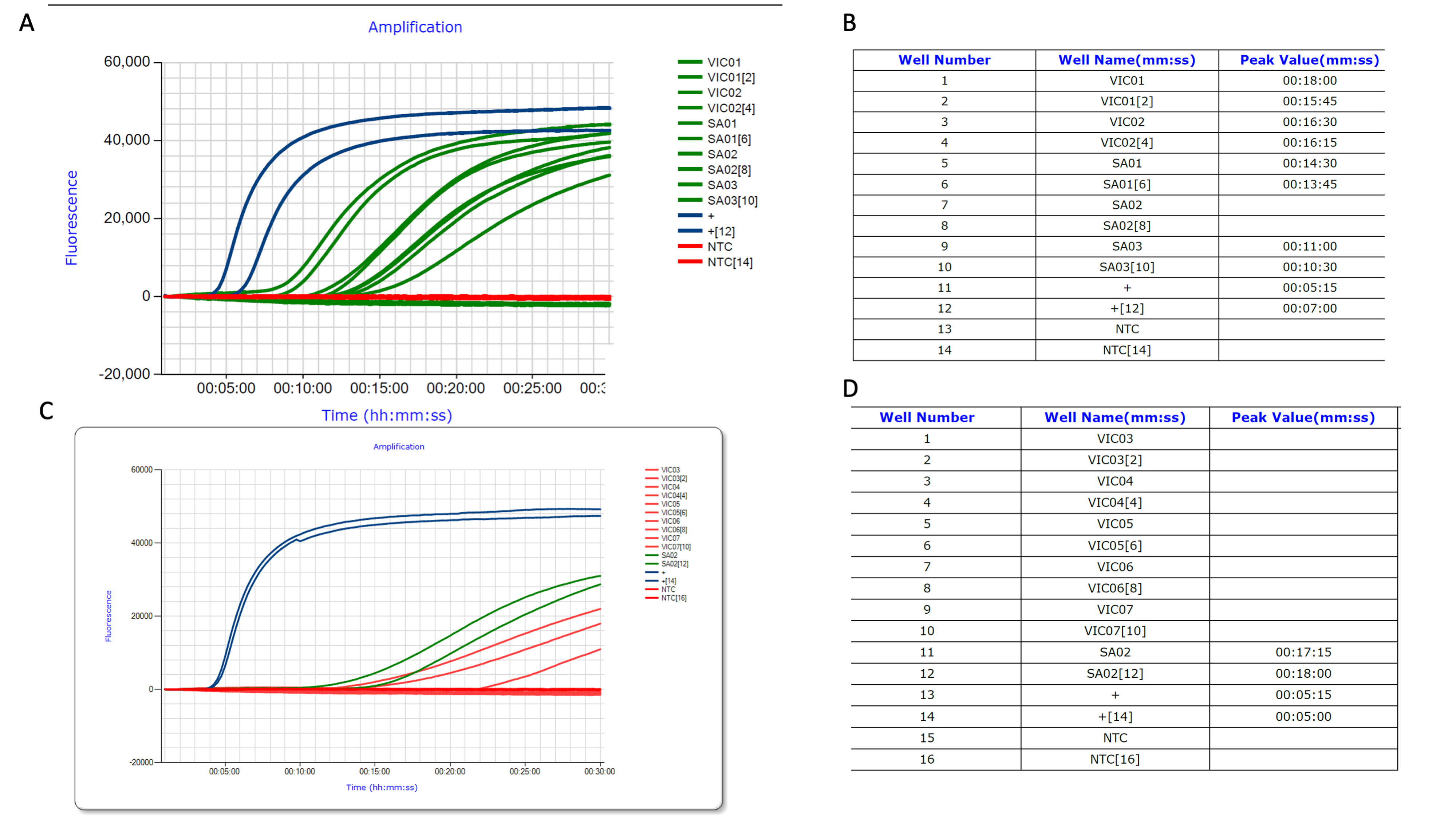

Supplement: Supplementary file 12 — (PNG 1150 kb) [file 436_2022_7520_Fig7_ESM.png]

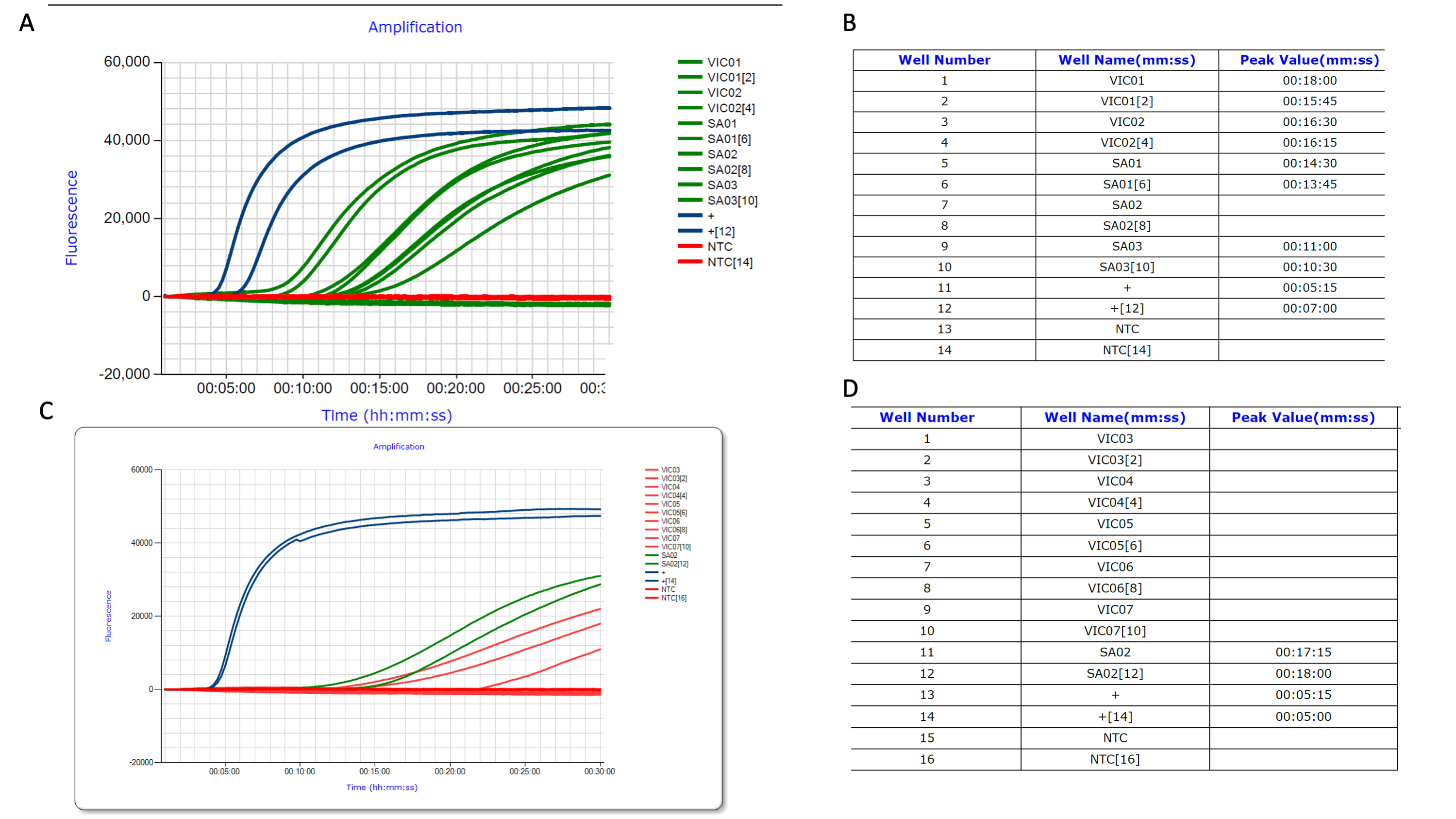

Supplement: Supplementary file 13 — High Resolution (TIFF 14826 kb) [file 436_2022_7520_MOESM7_ESM.tiff]
